# Estimating completeness of birth registration in South Africa, 1996 – 2011

**DOI:** 10.2471/BLT.18.222620

**Published:** 2019-05-28

**Authors:** Nadine Nannan, Robert Dorrington, Debbie Bradshaw

**Affiliations:** a Burden of Disease Research Unit, South Africa Medical Research Council, Francie van Zijl Drive, Parow valley, Cape Town, 7501, South Africa.; b Centre for Actuarial Research, University of Cape Town, Cape Town, South Africa.

## Abstract

**Objective:**

To estimate the completeness of live birth registration through South Africa’s civil registration and vital statistics system between 1996 and 2011.

**Methods:**

The number of births registered by the civil registration and vital statistics system was compared with independent estimates of the true number of births derived using: (i) the reverse survival method applied to 2011 census data; (ii) the application of estimated age-specific fertility rates to population estimates from censuses and surveys; and (iii) data from the public-sector district health information system.

**Findings:**

In 1996, an estimated 25% of births were registered within the calendar year of birth and 33% were registered before the end of the subsequent calendar year. By 2008, 76% of registrations occurred within the calendar year of birth, 84% occurred by the end of the following year and 90% occurred before the child’s fifth birthday. These improvements were seen in all provinces and differences in completeness between provinces narrowed markedly. Improvements in the completeness of registration coincided with government efforts to strengthen the system, new legislation on vital registration and the introduction of child support grants, which required birth certificates. Interprovincial migration of children influenced the completeness of registration in affected provinces. There was some terminological confusion among government agencies on defining the timeliness of registration and the year of birth.

**Conclusion:**

The completeness of birth registration in South Africa increased rapidly between 1996 and 2004. To allow international comparison, the method for measuring the completeness of birth registration needs to be standardized.

## Résumé

**Objectif:**

Estimer l'exhaustivité de l'enregistrement des naissances vivantes dans le système sud-africain d'enregistrement et de statistiques de l'état civil entre 1996 et 2011.

**Méthodes:**

Le nombre de naissances enregistrées par le système d'enregistrement et de statistiques de l'état civil a été comparé à des estimations indépendantes du nombre réel de naissances, obtenues grâce à: (i) la méthode des coefficients de survie inversée, appliquée aux données du recensement de 2011; (ii) l'application d'estimations du taux de fécondité par âge à des prévisions démographiques issues de recensements et d'enquêtes; (iii) des données du secteur public extraites du système d'information sanitaire des districts.

**Résultats:**

On estime qu'en 1996, 25% des naissances ont été enregistrées pendant l'année civile de la naissance et 33% avant la fin de l'année civile suivante. En 2008, 76% des enregistrements ont eu lieu pendant l'année civile de la naissance, 84% avant la fin de l'année suivante et 90% avant le cinquième anniversaire de l'enfant. Ces améliorations ont été observées dans toutes les provinces et les différencesentre provinces en matière d'exhaustivité ont sensiblement diminué. L'amélioration de l'exhaustivité des enregistrements a coïncidé avec les efforts du gouvernement pour renforcer le système, avec une nouvelle législation sur l'enregistrement des données d'état civil ainsi qu’avec la mise en place d'allocations d'entretien des enfants exigeant un certificat de naissance. La migration interprovinciale des enfants a influé sur l'exhaustivité des enregistrements dans les provinces concernées. Les agences gouvernementales ont connu une certaine confusion terminologique, s'agissant de définir le moment d'enregistrement et l'année de naissance.

**Conclusion:**

L'exhaustivité de l'enregistrement des naissances en Afrique du Sud s’est rapidement améliorée entre 1996 et 2004. Afin de permettre une comparaison internationale, il est nécessaire de standardiser la méthode employée pour mesurer l'exhaustivité de l'enregistrement des naissances.

## Resumen

**Objetivo:**

Estimar la integridad de la inscripción de los nacidos vivos mediante el sistema de registro civil y estadísticas vitales de Sudáfrica entre 1996 y 2011.

**Métodos:**

El número de nacimientos registrados por el sistema de registro civil y de estadísticas vitales se comparó con estimaciones independientes del número real de nacimientos mediante el uso de: (i) el método inverso de supervivencia aplicado a los datos del censo de 2011; (ii) la aplicación de tasas de fecundidad estimadas por edad a las estimaciones de población de los censos y encuestas; y (iii) los datos del sistema de información sanitaria del sector público de los distritos.

**Resultados:**

En 1996, se estimó que el 25 % de los nacimientos se inscribieron en el mismo año natural y el 33 % se inscribieron antes de que finalizara el año natural siguiente. En 2008, el 76 % de las inscripciones se produjeron a lo largo del mismo año natural, el 84 % al final del año siguiente y el 90 % antes de que el niño cumpliera cinco años. Estas mejoras se observaron en todas las provincias y las diferencias de integridad entre ellas se redujeron notablemente. Las mejoras en la integridad del registro coinciden con los esfuerzos del Gobierno por fortalecer el sistema, la nueva legislación sobre el registro civil y la introducción de subsidios de manutención infantil, que exigen certificados de nacimiento. La migración interprovincial de niños influyó en la integridad del registro en las provincias afectadas. Existe cierta confusión terminológica entre los organismos gubernamentales a la hora de definir el momento de la inscripción y el año de nacimiento.

**Conclusión:**

El número de nacimientos registrados en Sudáfrica aumentó rápidamente entre 1996 y 2004. Para permitir la comparación internacional, es necesario normalizar el método de medición de la integridad del registro de nacimientos.

## ملخص

**الغرض:**

تقدير اكتمال تسجيل المواليد الأحياء من خلال نظام التسجيل المدني والإحصاءات الحيوية في جنوب أفريقيا بين عامي 1996 و2011.

**الطريقة:**

تمت مقارنة أعداد المواليد المسجلين بواسطة نظام التسجيل المدني والإحصاءات الحيوية، بالتقديرات المستقلة للعدد الحقيقي للمواليد المشتق باستخدام:(1) الطريقة العكسية للبقاء على قيد الحياة، والمطبقة على بيانات التعداد لعام 2011؛ و(2) تطبيق معدلات الخصوبة المحددة حسب العمر، على التقديرات السكانية من التعدادات والمسوحات؛ و(3) البيانات من نظام المعلومات الصحية في القطاع العام.

**النتائج:**

في عام 1996، تم تسجيل ما يقدر بنحو 25% من المواليد في غضون سنة تقويمية للولادات، و33% تم تسجيلها قبل نهاية السنة التقويمية اللاحقة.بحلولعام 2008، حدثت 76% من التسجيلات خلال السنة التقويمية للولادة، وحدثت 84% منها بنهاية العام التالي، وحدثت 90% منها قبل عيد الميلاد الخامس للطفل.وظهرت هذه التحسينات في كل المقاطعات، كما تمت ملاحظة أن الاختلافات في الاكتمال بين المقاطعات كانت بسيطة.تزامنت التحسينات في اكتمال التسجيل مع جهود الحكومة لتقوية النظام، والتشريع الجديد بشأن التسجيل الحيوي، وطرح منح إعالة الأطفال، والتي تتطلب وجود شهادات الميلاد.أثرت هجرة الأطفال بين المقاطعات على اكتمال التسجيل في المقاطعات المعنية.كان هناك بعض الارتباك بخصوص المصطلحات بين الهيئات الحكومية بخصوص تحديد دقة توقيت التسجيل، وسنة الميلاد.

**الاستنتاج:**

تزايداكتمال تسجيل المواليد في جنوب إفريقيا بسرعة بين عامي 1996 و2004. للسماح بإجراء مقارنة دولية، تحتاج طريقة قياس اكتمال تسجيل المواليد للتوحيد القياسي.

## 摘要

**目的:**

旨在估计 1996 至 2011 年间通过南非户籍和生命统计体系进行出生人口登记的完成度。

**方法:**

将户籍和生命统计系统中登记的出生人数与推算得到的实际出生人数的独立评估数字进行对比，实际出生人数的独立评估数字通过如下方式获取：(i) 将反向幸存法应用于 2011 年人口普查数据；(ii) 将针对年龄的人口出生率估值应用于来自人口普查和调查的人口评估数；以及 (iii) 来自公共部门地区卫生信息系统的数据。

**结果:**

据估计，1996 年共有 25% 的出生人口在出生当年进行了登记，33% 的出生人口在下一个日历年度结束之前进行了登记。截至 2008 年，出生当年进行登记的比例为 76%，下一年结束前进行登记的比例为 84% ，儿童五岁前完成登记的比例达到 90%。以上改善在所有省份均有所呈现，且各省之间的完成度差异明显缩小。登记完成度方面的改善与政府在增强系统、针对出生登记制定新的立法以及儿童抚养补贴（需要出生证明）政策方面的工作相得益彰。儿童的跨省迁移会影响相关省份的出生登记完成度。在界定登记的及时性和出生年份方面，政府机构之间存在一些术语上的混淆。

**结论:**

南非出生登记完成度在 1996 年至 2004 年之间实现了显著提升。为了进行国际间比较，应对出生登记完成度衡量方法进行标准化。

## Резюме

**Цель:**

Оценка полноты данных при регистрации живорожденных в системе регистрации актов гражданского состояния и демографической статистики Южной Африки в период между 1996 и 2011 годом.

**Методы:**

Количество рождений, зафиксированных в системе регистрации актов гражданского состояния и демографической статистики, сравнивалось с независимой оценкой истинного количества рожденных детей, которая была получена с использованием: (I) обратного метода анализа доли доживающих до определенного возраста применительно к данным переписи населения на 2011 год; (ii) оценочного коэффициента фертильности (с учетом возрастных групп) для оценки популяции по данным переписей населения и опросов; (iii) данных информационных систем региональных учреждений общественного здравоохранения.

**Результаты:**

В 1996 году примерно 25% новорожденных регистрировались в течение календарного года их рождения, а 33% регистрировались до окончания следующего календарного года. К 2008 году 76% регистраций новорожденных приходились на календарный год рождения, 84% совершались к концу следующего года, а 90% детей оказывались зарегистрированными до пятого дня рождения. Такие улучшения отмечались во всех провинциях, и разница между полнотой регистрации в разных провинциях существенно сократилась. Улучшения в части полноты данных при регистрации совпали с усилиями правительства по укреплению системы, введением нового законодательства о регистрации актов гражданского состояния и субсидий на поддержку детей, для получения которых требовались свидетельства о рождении. В затронутых миграцией провинциях перемещение детей из одной провинции в другую влияло на полноту регистрации. Наблюдалось некоторое рассогласование в определении правительственными органами таких понятий, как «своевременная регистрация» и «год рождения».

**Вывод:**

Полнота данных регистрации рождений в Южной Африке резко увеличилась в период между 1996 и 2004 годом. Для сравнения этих данных с международными данными необходима стандартизация метода измерения полноты данных регистрации рождений.

## Introduction

Actions taken globally to achieve the millennium development goals highlighted the failure of civil registration and vital statistics systems in some low- and middle-income countries to record vital events accurately and to provide data essential for monitoring key child health indicators. The United Nations Secretary-General's Global Strategy for Women's and Children's Health 2016–2030 further emphasizes the importance of these systems for monitoring maternal and child health.^1^ Moreover, the sustainable development goals (SDGs) include the target of achieving universal birth registration by 2030. This target stems from the overarching objective of improving data systems for monitoring health-related indicators and reflects a desire to improve equity among population groups.^2^

Birth registration, which is enshrined in the United Nations Convention on the Rights of the Child,^3^ is fundamental to the legal recognition of human beings and, consequently, to their ability to secure a name and nationality. Statistically, it is defined as the continuous, permanent and universal recording of the occurrence and characteristics of births.^4^ Birth registration is a pivotal component of civil registration and vital statistics, and is key along with death registration, to monitoring trends in child mortality. Well-maintained registration systems provide essential data for assessing priority areas of population health. Less recognized are the links between birth registration and social development in modern societies,^5^^,^^6^ particularly in addressing poverty. In 2013, the United Nations Children's Fund (UNICEF) estimated that globally the births of only 65% of children younger than 5 years were registered.^4^ To draw attention to the importance of birth registration, UNICEF produced two publications on inequities and trends in birth registration and a handbook for those working on birth registration.^4^^,^^7^ In November 2016, the United Nations Population Division held an expert group meeting on evaluating the completeness and quality of vital statistics data.^8^

In the publication *Every child’s birth right*,^4^ which detailed national and regional trends in birth registration, UNICEF used data in an annual report from South Africa’s national statistical office to conclude that in 2011, the country achieved 95% completeness of birth registration within the first year of life. However, this estimate was derived by dividing the number of birth occurrences in 2012 by registrations in 2012, effectively it assessed the timing of registration rather than its completeness. Alternative estimates of completeness, based on different methods and data sources, are available for South Africa. According to national household surveys in 2008 and 2011, 11% of children younger than 3 years of age did not have a birth certificate.^9^^,^^10^ However, proof of registration was not sought in either survey. The Department of Home Affairs, which monitors babies registered within the year of birth, reported that the proportion was 90% in the financial year 2011 to 2012.^11^ Although the proportion is similar to that found in household surveys, like UNICEF’s estimate, it is a measure of registration timing rather than completeness.

Evidently, a standard measure or method for monitoring the completeness of birth registration is lacking. The World Health Organization defines completeness as “a measure of the extent to which births and deaths in a country in a given year are registered by the civil registration system.”^12^ Any measure of completeness therefore requires an independent estimate of the number of births or deaths. One concern is the need for a clear definition of the time frame, or the cut-off date, for registration. Current measures consider completeness within either 1, 3 or 5 years of birth.

To date, no estimate of the completeness of birth registration in South Africa has been based on estimates of the true number of births. The aims of this study therefore were to assess the completeness of birth registration in South Africa’s nine provinces between 1996 and 2011, with particular reference to the SDG’s focus on monitoring and reducing health inequities, and to propose a method for annual assessment.

## Methods

South Africa, an upper-middle-income country with a population of over 56 million individuals in 2016, has a well-established civil registration and vital statistics system, but still faces challenges in using the data to track births and deaths, and particularly, to estimate child mortality.^13^^,^^14^ The registration of births and deaths is governed by the Births and Deaths Registration Act of 1992,^15^ which has been amended several times, probably the most important amendment was the introduction of new birth and death notification forms in 1998.^16^ One crucial initiative for improving timely birth registration was providing new mothers with information about the process during antenatal care visits.^16^ In addition, birth notification forms were made available at state facilities at the time of delivery. In South Africa, births are registered by the Department of Home Affairs using information provided on the birth notification forms. The information is captured on the National Population Register (i.e. civil registration) and these data are submitted to the national statistical office (Statistics South Africa), which compiles annual reports on birth statistics.^17^

We used unit record data to create tables of births by year of occurrence and year of registration. The statistical office defines late registration as registration after the calendar year of birth (Statistics South Africa, unpublished data, 2015), with the calendar year of registration closing at the end of February of the following year. We regarded births registered before the end of February of the calendar year following the calendar year of birth as being registered in the year of birth and births registered in the following calendar year as being registered in the year of birth + 1, and so on.

### Estimating the number of births

First, estimates of the true number of births between 1996 and 2010 were derived using the reverse survival method from the enumerated South African-born population recorded in the 2011 census, allowing for interprovincial migration between 2001 and 2011.^18^ Specifically, data on children, by age and province of birth, were used. The method followed age cohorts in the recorded population back to their year of birth by applying appropriate age, sex and time period-specific probabilities of surviving from birth to age *x*. Hence, the number of children aged 0 to 15 years recorded in the 2011 census were projected backward to estimate the number of births for each age cohort using the life table measures, 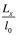, where *L_x_*is the number of person-years lived between age and age *x + 1* (*x* can vary from 0 to 15) recorded in the cohort life table, of which *l_0 _*is the radix (i.e. the number of births in the age cohort). Estimating the number of births in any year involved the following stages: (i) age cohort survival probabilities to 2011, which were applicable to births over the past 15 years, were derived using the Actuarial Society of South Africa’s 2008 population projection model with the migration assumptions set to zero;^19^ (ii) each survival factor was derived by dividing the number of individuals aged *x* last birthday in 2011 by the number of births these individuals originated from in the year in which they were born; (iii) each survival factor was then used to estimate the number of births in year *y* (*B_y_*) by dividing the number in a particular age cohort recorded in the 2011 census (in the country or province) by the appropriate survival factor. For example, the number of births in the year starting 10 October 2010 (*B_2010_*) was derived by dividing the number of children under 1 year of age (born in the area of interest) in the 2011 census () by the survival factor for this age cohort from the model, 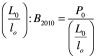
(iv) as censuses take place in October and the numbers derived from census data are for the 12 months before the date of the census, the number of births in a calendar year was calculated by apportioning births over census years. For example, the number of births in calendar year 2010 was 0.7726 × *B_2010_* plus 0.2274 × *B_2009_*; and (v) the number of births in different provinces in each year was scaled to ensure that the sum of the births in all provinces equalled the national total.

Second, an alternative estimate of the number of births against which the completeness of birth registration could be evaluated was derived using estimated total fertility rates. We based these rates on national and provincial fertility research in South Africa between 1996 and 2011.^20^ The researchers initially derived annual estimates of age-specific and total fertility from summary birth history data from the 1996, 2001 and 2011 censuses, as well as from a 2007 community survey and from full birth history data from the 1998 Demographic and Health Survey. We assumed that fertility rates changed linearly between data collection points. We then obtained estimates of total fertility by calculating the number of births occurring in any year by applying age-specific fertility rates to annual estimates of the population of women by age. We used age-specific fertility rates to derive the number of births occurring in different years for comparison with the number derived by projecting backward from the population recorded in the 2011 census.

Third, we estimated the number of births between 2004 and 2012 using routine health statistics collected by the District Health Information System from public sector health facilities in all nine provinces. In 1994, the introduction of free health care for mothers and children up to 6 years of age led to a substantial increase in the number of births taking place in the public sector.^21^ Nevertheless, District Health Information System data had to be adjusted for births that occurred in private facilities or at home. A review of the 1998 and 2003 South African Demographic and Health Surveys and of the 2012 South African National HIV Prevalence, Incidence and Behaviour Survey revealed that insufficient data were available to provide robust estimates of the proportion of births that occurred either in the private sector or at home.^22^^–^^24^ However, they did show that births in private facilities were more common in richer, more urban provinces (e.g. Gauteng and the Western Cape) than in poorer, more rural provinces (e.g. the Eastern Cape, Mpumalanga and Limpopo), where the proportion of home births was greater. We used the proportion of children younger than 1 year of age who were covered by medical aid schemes or health insurance, as reported in General Household Surveys,^25^ as a proxy for the proportion of births in the private sector: the average was 11.9% between 2004 and 2012. We estimated the annual proportion of home births in each province from the proportion of rural residents because we found a strong correlation (i.e. *R^2^* = 0.74) between the proportion of rural residents reported in the 1996 census in a province and the proportion of home births recorded in the 1998 Demographic and Health Survey in that province. We used the following regression equation to estimate the percentage of home births in province *i* (*hb_i_*) from the percentage of home births recorded nationally in 2010 (*HB*):
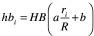
(1)where *r_i_* is the percentage of the population in province *i* that was rural, *R* is the percentage of the national population that was rural in 2010, and *a* and *b* are regression coefficients. As far as possible, we followed the Guidelines for Accurate and Transparent Health Estimates Reporting.^26^

## Results

**Fig. 1:**
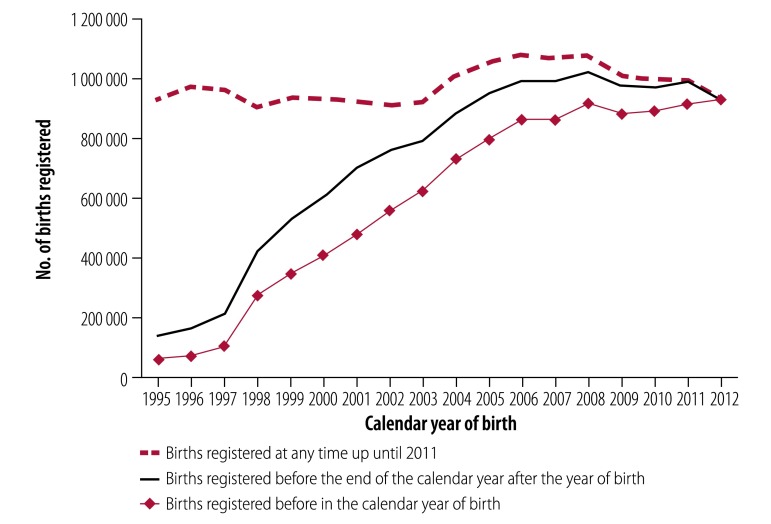
Birth registration, by calendar year of birth and time of registration, South Africa, 1995–2012

Notes: The figure shows births reported to the national statistical office. The calendar year of registration closed at the end of February of the following year.

Fig. 1 shows the registered number of births reported annually to the national statistical office between 1995 and 2012. The number increased from 924 207 birth registrations in 1995 and peaked at around 1 080 000 birth registrations between 2006 and 2008 before declining in recent years due to late registrations not yet included in the data. Between 1997 and 2005, there was a substantial improvement in the promptness of birth registration, which continued to 2008, after which it remained stable.

**Fig. 2:**
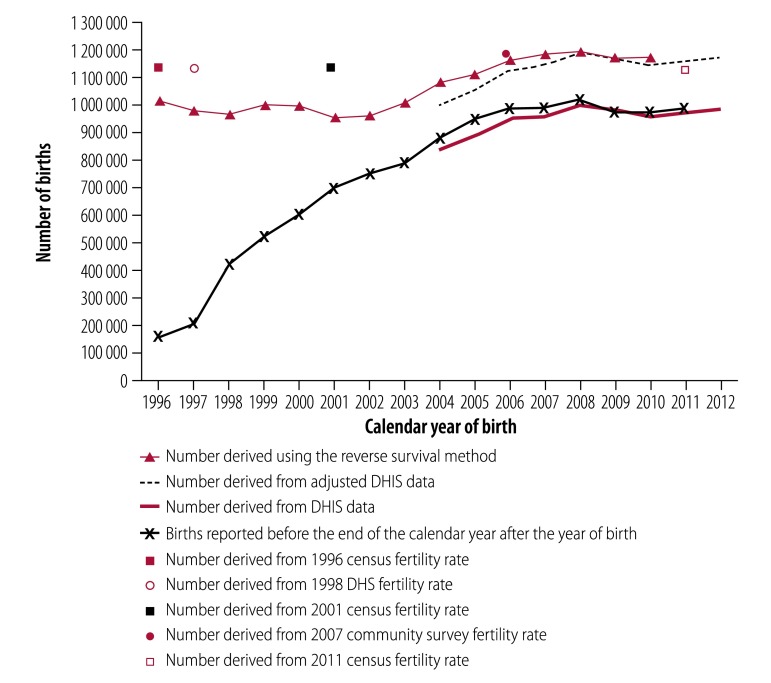
Estimated and reported births, South Africa, 1996–2012

DHIS: District Health Information System; DHS: Demographic and Health Survey.

Notes: The number of births was estimated using: (i) the reverse survival method applied to data from the 2011 census; (ii) fertility rates; and (iii) routine health statistics from the District Health Information System (DHIS), with and without adjustment for births in private health facilities and at home. Reported births comprised those reported to the national statistical office before the end of the calendar year after the year of birth. The calendar year of registration closed at the end of February of the following year.

Fig. 2 shows the number of births between 1996 and 2012 estimated using the reverse survival method, fertility rates and routine health statistics. The number estimated using the reverse survival method was around 1 million births annually until about 2003, then increased to about 1.2 million in 2007 and 2008 and then levelled off. These estimates were very close to the estimates from the full census population count, which included people not born in South Africa (data available from the corresponding author). Fig. 2 also shows point estimates of the annual number of births derived using fertility rates reported by women in censuses and surveys (Table 1). The estimates were fairly constant, ranging from 1 124 000 estimated number of births in 2011 to 1 168 355 in 2001. Estimates derived from routine health statistics are practically identical to those obtained using the reverse survival method from 2008 onwards after adjustment for births in private health facilities and at home (which resulted in an overall increase of 16.3%). These estimates also plateau between 2008 and 2012. For comparison, Fig. 2 also shows births registered by the civil registration and the vital statistics system in the year after the calendar year of birth. Despite improvements over the 17-year period, persistent under-registration is evident.

**Table 1:** Number of births estimated using fertility rates derived from censuses and surveys, South Africa, 1996–2011

Year	Data source	Derivation of age-specific fertility rates	Estimated number of births
1996	Census	Moultrie and Dorrington 2004^27^	1 130 571
1998	Demographic and Health Survey	Moultrie and Dorrington 2004^27^	1 126 310
2001	Census	Moultrie and Dorrington 2004^27^	1 128 238
2007	Community survey	Darikwa^28^	1 168 355
2011	Census	Dorrington and Moultrie^20^	1 124 000

### Completeness of registration

**Fig. 3:**
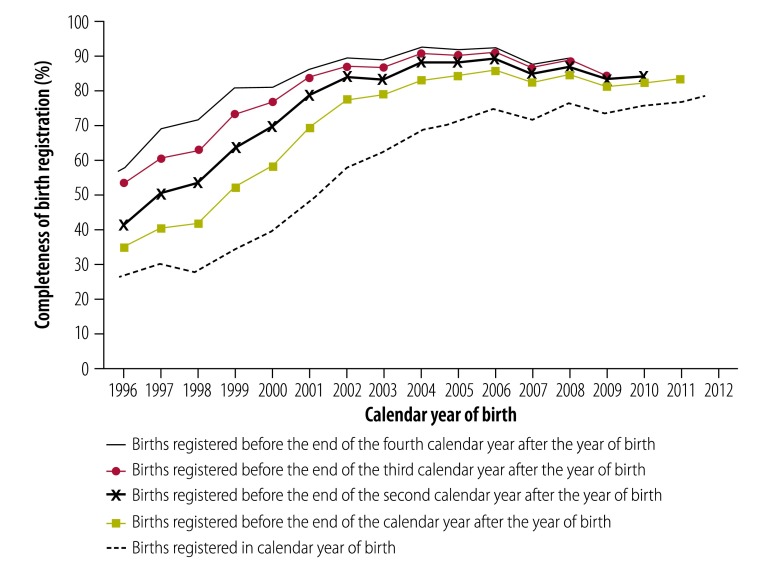
Completeness of birth registration by age, South Africa, 1996–2012

Notes: Completeness was assessed using the number of births in any year derived from 2011 census data using the reverse survival method. The calendar year of registration closed at the end of February of the following year.

Fig. 3 shows the estimated completeness of birth registration data between 1996 and 2011, by the year after birth in which registration occurred, as calculated using the estimated number of births derived by the reverse survival method. The completeness of registration between birth and the child’s fifth birthday improved substantially between 1995 and 2004, with the greatest improvement occurring for children younger than 1 year of age. In 1996, an estimated 25% of registrations took place in the calendar year of birth and 33% took place before the end of the calendar year after the year of birth. By 2008, these proportions had improved to 76% and 84% respectively. In 2004 and 2005, about 92% of births were registered before the end of the fourth year after the year of birth.

**Fig. 4:**
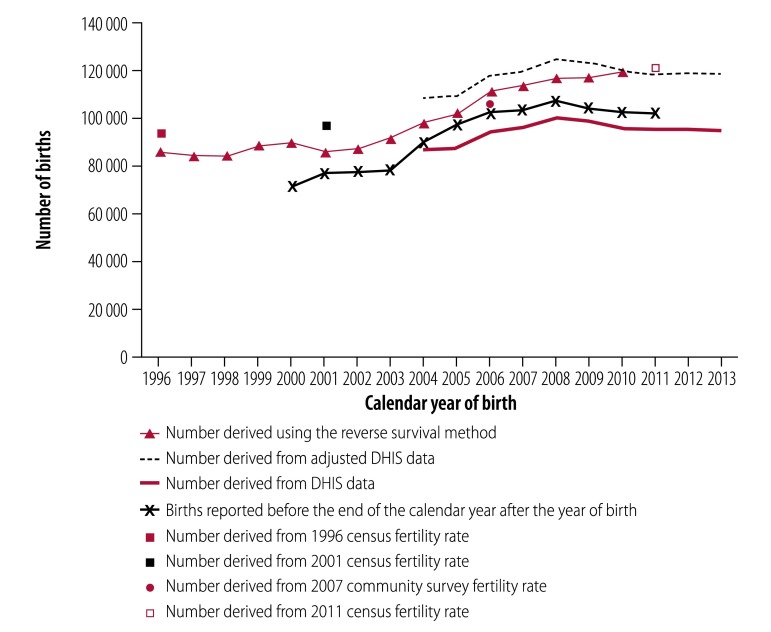
Estimated and reported births, Western Cape Province, South Africa, 1996–2013

**Fig. 5:**
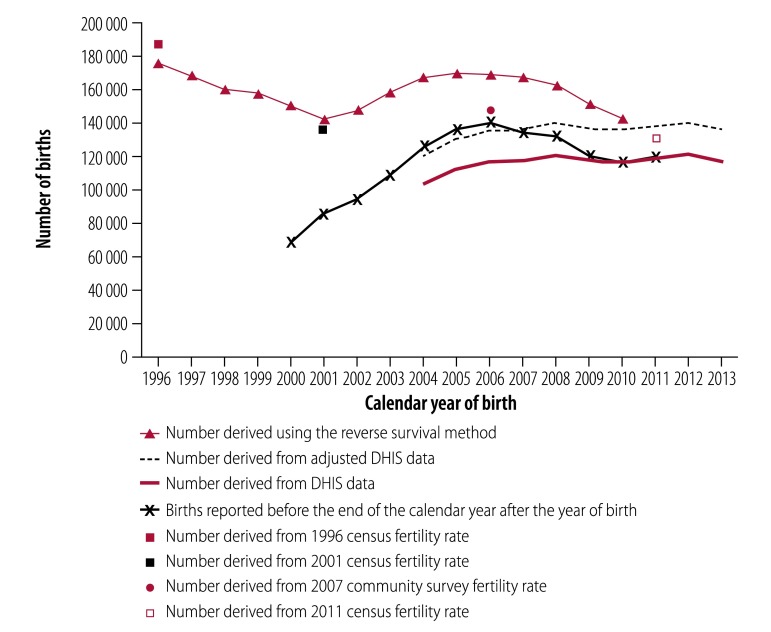
Estimated and reported births, Eastern Cape Province, South Africa, 1996–2013

Table 2 shows the completeness of birth registration before the end of the year after the calendar year of birth in individual provinces derived using both the reverse survival method and routine health statistics adjusted for births in private health facilities and at home. Overall completeness improved considerably between 2001 and 2011, and differences between different geographical settings narrowed over time. Surprising findings were that completeness was: (i) greater than the national average in the Free State and the North West; (ii) lowest in Gauteng; and (iii) greater than 100% in the North West in some years, when calculated using the reverse survival method. These anomalies may have occurred because children migrated early in life from their province of birth to the province in which their birth was registered. For example, differences in the estimated and reported number of births between the Eastern Cape and the Western Cape suggest that migration between these provinces may have occurred. The estimate for the Western Cape derived using adjusted routine health statistics was higher than that derived using the reverse survival method (Fig. 4), whereas the reverse was observed in the Eastern Cape (Fig. 5). Some mothers from the Eastern Cape may have travelled to the Western Cape to deliver their babies.

**Table 2:** Estimated completeness of birth registration before the end of the calendar year after the year of birth, by province and data source, South Africa, 2001–2011

Province	Data source^a^	Completeness of birth registration, %										
		2001	2002	2003	2004	2005	2006	2007	2008	2009	2010	2011
Eastern Cape	Census	60	64	69	75	80	83	80	81	80	82	ND
	DHIS	ND	ND	ND	122	121	120	115	111	104	102	94
Free State	Census	66	74	79	80	83	86	82	86	83	86	ND
	DHIS	ND	ND	ND	101	103	105	105	107	107	112	112
Gauteng	Census	84	87	85	90	89	85	83	82	79	76	ND
	DHIS	ND	ND	ND	96	97	98	97	97	93	98	95
KwaZulu-Natal	Census	67	81	77	76	80	87	84	86	82	84	ND
	DHIS	ND	ND	ND	100	105	110	110	108	107	109	108
Limpopo	Census	65	67	70	74	77	77	78	82	81	82	ND
	DHIS	ND	ND	ND	98	97	96	96	96	95	99	99
Mpumalanga	Census	57	67	76	83	83	85	86	88	84	86	ND
	DHIS	ND	ND	ND	113	110	110	111	110	110	113	113
Northern Cape	Census	72	76	79	84	88	89	90	93	90	91	ND
	DHIS	ND	ND	ND	105	107	108	111	112	113	113	114
North West	Census	78	83	90	93	101	99	100	103	100	97	ND
	DHIS	ND	ND	ND	126	130	132	134	132	132	137	134
Western Cape	Census	85	90	87	95	97	98	92	94	89	87	ND
	DHIS	ND	ND	ND	104	111	112	111	110	111	115	114
Nationally	Census	73	79	79	82	86	86	84	86	84	83	ND
	DHIS	ND	ND	ND	105	107	105	103	102	99	101	102

DHIS: District Health Information System; ND: not determined.

^a^ The completeness of birth registration before the end of the calendar year after the year of birth was derived from 2011 census data using the reverse survival method and from routine health statistics in the District Health Information System (DHIS) adjusted for births in private health facilities and at home. The calendar year of registration closed at the end of February of the following year.

DHIS: District Health Information System

Notes: The number of births was estimated using: (i) the reverse survival method applied to data from the 2011 census; (ii) fertility rates; and (iii) routine health statistics from the District Health Information System (DHIS), with and without adjustment for births in private health facilities and at home. Reported births comprised those reported to the national statistical office before the end of the calendar year after the year of birth. The calendar year of registration closed at the end of February of the following year.

DHIS: District Health Information System

Notes: The number of births was estimated using: (i) the reverse survival method applied to data from the 2011 census; (ii) fertility rates; and (iii) routine health statistics from the District Health Information System (DHIS), with and without adjustment for births in private health facilities and at home. Reported births comprised those reported to the national statistical office before the end of the calendar year after the year of birth. The calendar year of registration closed at the end of February of the following year.

## Discussion

Our study illustrates the usefulness of a robust method for assessing the completeness of birth registration that benefits from considering multiple data sets, particularly less frequently used data sets. We found that the completeness of birth registration in South Africa increased rapidly between 1995 and 2004 and that by 2011, 83% of registrations occurred before the end of the year after the calendar year of birth. This improvement was seen in all provinces and the relative difference between provinces narrowed markedly. The increase in birth registration observed after 1995 coincided with the introduction of amendments to the 1992 Birth and Deaths Registration Act that extended it to include former homelands and with efforts to strengthen vital registration. In addition, the introduction of child support grants in 1998 also played a role because the primary eligibility requirement was that the child’s birth had to be registered.^29^

Our estimate of the completeness of birth registration differed somewhat from UNICEF’s finding in 2011 that 95% of births in South Africa were registered within the first year of life.^4^ The main reason for this difference was the method used to assess completeness. We compared the number of births registered by a given age with an independent estimate of the total number of births in the relevant year. In contrast, UNICEF looked at the proportion of registered births that were registered within the first year of life. In 2013, UNICEF reported a variation globally: in some countries, measures of completeness were based on surveys, whereas in other countries they were based on the analysis of vital registration data. The method used to measure the completeness of birth registration needs to be standardized and international comparisons should use consistent and well defined assessment criteria and methods of evaluation.

### Limitations

The accuracy of the figures we derived for the estimated numbers of births using the reverse survival method was highly dependent on the accuracy of the enumeration of children in the 2011 census. We believe the 2011 census count was accurate because the figures were comparable with subsequent assessments of the number of older children in school and with the number of survivors of children enumerated in previous censuses.^20^ Furthermore, the numbers of births calculated by the reverse survival method using 2011 census data corresponded to the numbers estimated using fertility rates and adjusted routine health statistics.

Another limitation is that our analysis assessed the completeness of registration according to the number of complete years after the calendar year of birth because the exact date of registration was not available as part of the unit record data. Consequently, estimates of completeness according to the exact age of the child, which are arguably more desirable, were approximate. Second, the migration of young children after birth made it difficult to interpret the completeness of birth registration in individual provinces. Nevertheless, completeness improved in all provinces and we could use data on births collected by the District Health Information System to monitor registration. Finally, because of the complexity of using multiple data sources to arrive at our estimates, we did not quantify uncertainty in completeness estimates or undertake a sensitivity analysis.

Despite the concerted effort of the South African government to improve the civil registration and vital statistics system, there remain obstacles that prevent approximately 20% of births being registered by parents within the year of birth. Insight into what these obstacles are and which communities are most affected are needed. In addition, our assessment of the completeness of birth registration identified some terminological confusion among government agencies on definitions of the timeliness of registration and the year of birth. Although any definition of the completeness of birth registration should compare the number of registered births with the actual number of births, definitions generally do not mention the timing of registration, which could be within 30 days or 12 months or before the age of 5 years. Indicators should therefore be clearly described and there should be a general agreement on standard measures.

The commendable improvement in birth registration in South Africa can be attributed to the government’s commitment to improving the civil registration and vital statistics system, to changes in legislation around vital registration and to particular policies, such as cash transfers directed at the welfare of children. Evaluating the impact of these interventions would help other countries develop policies and strategies for improving birth registration.
